# Coaching and talent development in esports: a theoretical framework and suggestions for future research

**DOI:** 10.3389/fpsyg.2023.1191801

**Published:** 2023-05-10

**Authors:** Kabir Bubna, Michael Geoffrey Trotter, Matthew Watson, Remco Polman

**Affiliations:** ^1^The International Federation of Esports Coaches (IFoEC), London, United Kingdom; ^2^Department of Psychology, Umeå University, Umeå, Sweden; ^3^Department of Performance Psychology, German Sport University Cologne, Cologne, Germany; ^4^Institute of Health and Wellbeing, Federation University Australia, Berwick, VIC, Australia

**Keywords:** skill acquisition, esports, coaching, ecological dynamic approach, talent development, constraints, affordances (ecological psychology)

## Abstract

Esports is a growing phenomenon that is capturing the attention of individuals worldwide, and has grown to provide professional and lucrative careers for those who reach the upper echelons. One question that arises, is how esports athletes develop the necessary skills required to improve and compete. This perspective piece opens the door to skill acquisition within esports and how research through an ecological approach can benefit researchers and practitioners as they understand the various perception-action couplings and decision-making challenges faced by esports athletes. We will identify and discuss what constraints look like in esports, the role of affordances, and theorize the implementation of a constraints-led approach in contrasting esports genres. As esports is technology-heavy in nature and generally sedentary, the use of eye-tracking technology is argued to represent an effective method to better understand perceptual attunement between individuals and teams. Future research into skill acquisition in esports is needed to develop a clearer picture of what makes the greatest esports player so great, and how newer players can be developed effectively.

## 1. Introduction

Esports has grown over the past couple of years to the point the International Olympic Committee is looking to host its first Esports week in Singapore in the summer of 2023. In addition, the 2022 Asian Games in Hangzhou was the first international event that featured esports as a medal sport (Watson et al., 2022). Furthermore, to date, over 400 professional sporting organizations have expanded their operations to include esports (Scholz et al., 2021). To this end, it is important to recognize that there are different esports genres. These include first-person shooters (FPS; e.g., Counter-Strike: Global Offensive (CS:GO), Valorant, Call of Duty) and massive online battle arenas (MOBA: e.g., League of Legends (LoL), Dota2). However, across genres, research has indicated that key stakeholders (i.e., professional players and teams) report dissatisfaction with their training and development processes (Abbott et al., 2022; Sabtan et al., 2022). Furthermore, it is common for high turnover within rosters in esports (LeNorgant, 2019) which restricts talent development and can impede on the coaching process. The aim of the present paper is to provide an overview of current practices and to draw attention toward future research embedded within skill acquisition with hopes to find answers.

This perspective piece will adopt the definition from Pedraza-Ramirez et al. (2020): “Esports is the casual or organized competitive activity of playing specific video games that provide a professional/and or personal development to the player … Consistent engagement within this structure allows individuals to develop fine-motor coordination, and perceptual-cognitive skills at all levels of competition” (p. 6).

There has been a rapid increase in academic research in esports (see Reitman et al., 2020 for a review) across multiple domains including business, marketing, and sport psychology. Beyond the need for physical (e.g., posture, physical fitness; Trotter et al., 2020; Migliore et al., 2021), nutritional (Ribeiro et al., 2021), and mental/psychological preparation (e.g., emotional regulation; Poulus et al., 2022), there is value in developing an objective understanding of skill acquisition in sports. However, no research has explicitly explored the domain of skill acquisition. Such research would help in understanding the components that are essential to develop and execute skills seen in high-performance environments.

Skill acquisition research has predominantly been conducted across a variety of traditional sports, such as football and tennis (Reid et al., 2007; Football Federation Australia, 2013; Farrow and Robertson, 2017), and it may prove useful to study skill acquisition and development of expertise in professional gamers as the industry continues to grow and to support coaching practice within a novel context. Through interaction with work done in skill acquisition, research can uncover what is relevant and unique in the development of e-athletes, and can use empirically supported strategies to create tailored and/or individualized programs to develop and sustain athletes at all levels of participation from grassroots to professional.

## 2. What is skill and skill acquisition?

The term skill can be defined as a player’s ability to appropriately identify, organize, and execute an action or pattern of behavior with effectiveness, consistency, and efficiency to solve a task (Williams et al., 2003). Skill acquisition is the field that understands the development of humans and expertise through an understanding of different sports science disciplines such as biomechanics, motor learning and control (Lindinger et al., 2022), sports psychology (cognitive capabilities) and pedagogy (Malhotra et al., 2022). This informs and supports the practice of current agents in the field of talent development (i.e., coaches; Hodges and Williams, 2019; Baker et al., 2021).

A large part of understanding the development of expertise in athletes lies in understanding the practice activities athletes engage in (e.g., frequency, practice structure, and duration; Ericsson, 2006; Côté et al., 2007). As a result, a large part of skill acquisition and talent development research has focused on what type of practice environment yields the greatest results (Ericsson et al., 1993). A predominant theoretical framework within skill acquisition is ecological dynamics. Ecological dynamics combines the work of ecological psychology (understanding the demands of the environment) and dynamical systems (mathematical understanding of the organism; Seifert and Davids, 2017). The combination of these frameworks views functional human movement as the result of an entwined and cyclical relationship between an organism and its environment (perception-action couplings; Handford et al., 1997).

## 3. Skill development and current esports research

Current research advocates for athletes to engage with deliberate and purposeful practice environments for skill development to be enhanced (Charness et al., 2005; Ericsson and Harwell, 2019; Farrow et al., 2013). Ericsson (2021) sets out five criteria that helps practitioners develop and integrate deliberate practice within their context: (1) clear session intention, (2) performer can complete the task individually, and is beneficial to their development, (3) provision of immediate and actionable feedback is available, (4) the performer is afforded multiple attempts at the practice task, and (5) the coach/teacher can oversee the development of future tasks to align with the needs of the performer.

More recently, a small number of studies have started investigating concepts related to skill acquisition and coaching practice in esports with are loosely aligned to Ericsson’s criteria. Pluss et al. (2021) conducted a study that investigated the effect practice quantity had on in-game performance within an eight-week span prior to competition. This study concluded that, at the professional level of participation, the quantity of practice explains only a very small fraction of in-game performance. However, it noted that there is a need for deliberate and purposeful practice to reach the upper echelons of competition. To build on this study Pluss et al. (2022) conducted a longitudinal analysis of practice quantity over a 52-week period, and analyzed practice behaviors between professional and semi-professional teams. Although the results were similar and showed a weak correlation between practice quantity and the development of expertise, it did suggest that players could invest up to 16,000+ hours over a 10-year period, with professional teams training more frequently than their semi-professional counterparts. This highlights the need to find more effective practice solutions and to prevent unhealthy practice behaviors, negative lifestyle outcomes, and burnout associated with esports (DiFrancisco-Donoghue et al., 2019; Salo and Information Resources Management Association (IRMA), 2021). Results and suggestions from the studies by Pluss et al. also align with the findings of a study conducted by Abbott et al. (2022) which investigated the perceived effectiveness of practice in LoL. Results indicated that players evidenced a lack of perceived effectiveness within their current training environment and believed that the predominant grind (i.e., a high quantity of games) approach was not the most effective method for the development and achievement of expertise.

## 4. An ecological approach to skill acquisition

Adopting an ecological approach (Gibson, 1979) can be beneficial in understanding and informing future research investigating skill acquisition and the development of expertise in esports athletes. Through an application of this perspective, skill acquisition and subsequent talent development can be viewed as the product of athlete-environment interactions and can hold the answers to developing better quality practice environments. This can be achieved through an understanding of key components within the ecological rationale like affordances, constraints, and a coaching framework known as the constraints-led approach.

### 4.1. Affordances in esports

Gibson (1979) states that an individual during performance is consistently surrounded by different modalities of information (i.e., optical, acoustic, and proprioceptive). This creates boundaries that influence the performer’s intention, coordination, and decision-making and is better known as the term *affordances* (Davids et al., 2013). An understanding of affordances and how it guides behavior is crucial. This results in skill adaptation, the process which athletes undergo to attain expertise in their chosen sport (Renshaw et al., 2019). Skill adaptation can be defined as the establishment of adaptive, and functional fit of movement solutions between the individual and their environment (Araújo and Davids, 2011). It is important to develop adaptable skills as even though the performance outcomes can be met, the process of perception-action of relevant affordances can be unpredictable as the environment changes, allowing different movement solutions and patterns to govern performance goals. Importantly, this notion of skill adaptability is also related to Bernstein ideas on the degrees of freedom problem in motor control.

Even within a single genre, games can be vastly different in the affordances within the game due to having different maps, characters, and in-game events/mechanics. However, all these games will feature comprehensive heads-up displays (HUDs) which include information pertaining to their character (i.e., health, resources, ammunition [FPS]), the two teams (friendly and opponents), a minimap, all of which can provide useful information to players as they compete. For example, the minimap will provide real-time information regarding the location of teammates and can provide the location of the opposition. Perception of this spatial constraint can inform the future movements of the individuals’ character to support allies or deny opportunities for enemy movements.

### 4.2. Constraints in esports

There is also an argument to be made that affordances are closely related to the constraints that individuals may face through the individual-character (FPS) or individual-champion (MOBA) relationship. First developed by Newell (1986), constraints are categorized into three different domains; (i) organism/individual, (ii) task, and (iii) environment.

**Figure 1:**
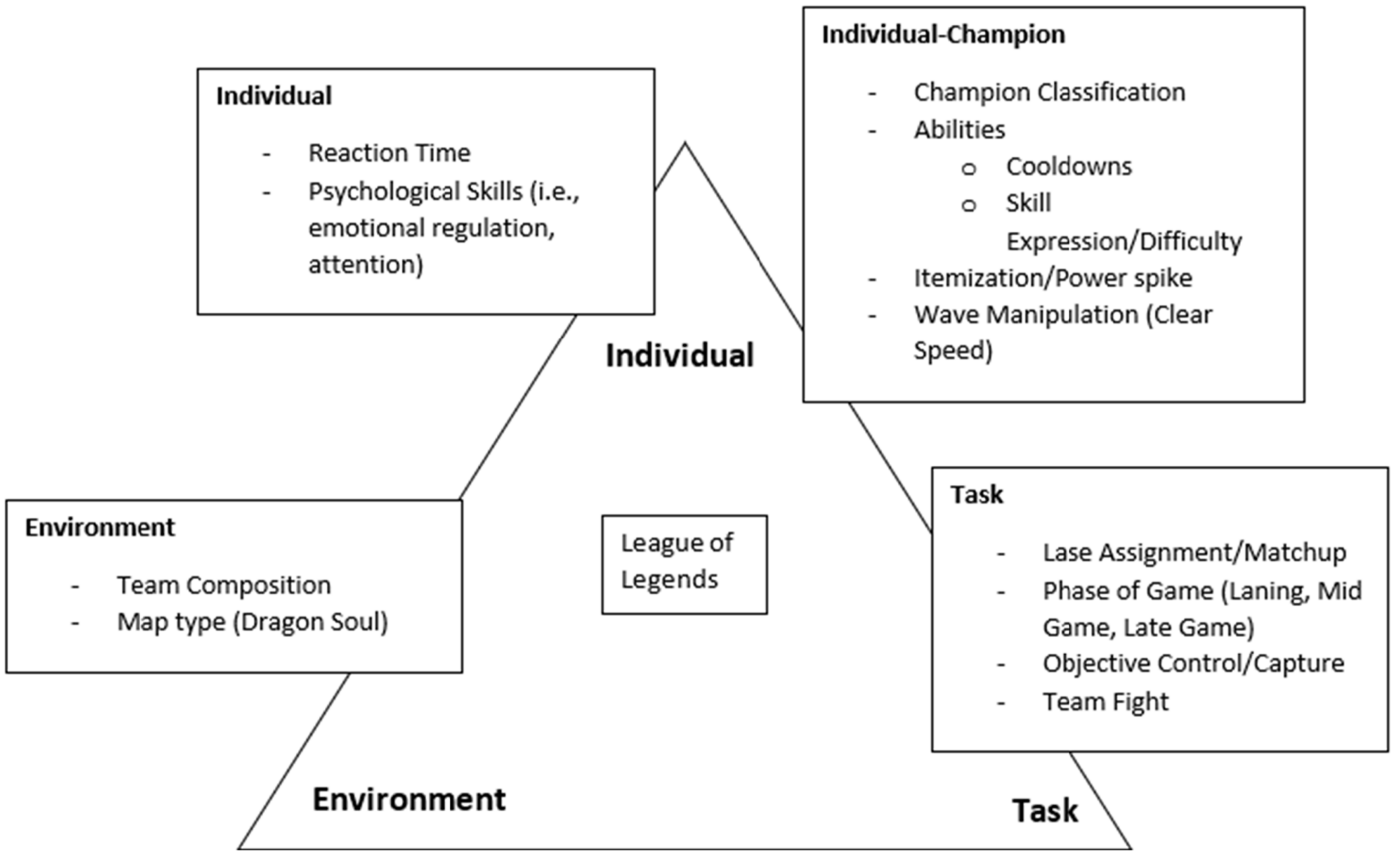
Adapting Newells’ (1986) model of interacting constraints to League of Legends.

Figure 1 provides an example of how LoL can be applied to Newells’ model of interacting constraints to identify the constraints that are relevant. This figure was adapted from Ziv (2023), where an ecological approach was applied to racecar driving. LoL is a highly complex game, not only through the various mechanisms needed to achieve successful performance but also fields a roster of 162 champions (at the time of writing). To be successful on a champion, the player must be familiar with their kit (champion abilities) and limits (individual constraints), and interactions with other champions (allies and opponents; environment constraint) within the game, as having different champions will provide different affordances which can then change the various decision-making strategies and tactics used to accomplish the outcome of the game (task constraints).

However, in FPS games, environmental constraints hold larger value, as during competition matches will be conducted on different maps, therefore players and teams are required to have in-depth knowledge of the layout, lines of sights, and key areas of control over a variety of different maps.

### 4.3. Applying the constraint-led approach within esports

With its roots based in the ED rationale, the constraint-led approach (CLA) framework can inform the development of practice design (Davids et al., 2008; Renshaw et al., 2019) although needs to be investigated in the esports contexts. Focusing on learning and the development of expertise through exploration and guided discovery (Chemero, 2009, 2013), CLA helps practitioners to identify key affordances within the practice environment and use of task, environment, and/or individual constraints (boundaries; Gray, 2018) to attune the attention of performers, therefore inviting them to interact with the intended behaviors (Rietveld and Kiverstein, 2014). To effectively implement CLA coaches are provided with criteria similar to the deliberate practice framework, known as the environment design principles (EDP): (1) Session Intention, (2) Constrain-to-afford, (3) Representative Learning Design, and (4) Repetition without Repetition. A thorough understanding of these principles allows practitioners to maintain action fidelity through representative practice (practicing how you play; Pinder et al., 2011; Seifert et al., 2013), while exposing the athletes to a variety of situations to develop adaptable movement solutions (Roberts et al., 2019; Travassos et al., 2012).

However, it is important to note that some genres of esports such as FPS (e.g., Counter-Strike: Global Offensive; CS:GO) allow users to code and modify games to a greater extent in order to create custom maps, game modes, and rules which better align with the nature of CLA, Other genres, such as multiplayer online battle arena (e.g., League of Legends) do not currently have the ability to manipulate the environment to the same degree, forcing users who wish to utilize a CLA to be more creative (refer to Appendix 1).

## 5. What is next for esports research?

Each individual esport provides ample research opportunity for an ecological investigation into skill acquisition and the attainment of expert performance. Studies such as Sharpe et al. (2023) in CS:GO represent a first step toward developing a comprehensive understanding of the components of performance in esports.

Although research has started to investigate the relationship between practice quantity and the development of expertise, there are larger questions still left unanswered when trying to understand skill acquisition and talent development in esports. It is increasingly important to continue to develop the depth and breadth of this scope of research as it can suitably offer solutions and support coaching practice in the process of creating more effective training methods with higher quality training processes. In doing so, research can also investigate how/if CLA can be effectively implemented across a variety of esport genres to enhance training methods.

We suggest three studies below, which are viable directions for future research that relate to an ecological approach to skill acquisition and also the implementation for CLA in esports.

### 5.1. Perceptual attunement between novices and experts in esports

Perceptual attunement is related to the difference between experts and novices in the information they perceive and value to perform a task (Fajen et al., 2008; Brand and de Oliveira, 2017). Within esports, athletes must progress up a ranking system which is seen to define their proficiency within the game (Pedraza-Ramirez et al., 2020), and within the current esports ecosystem, there are a plethora of organizations that provide coaching to help athletes develop their skills to climb the ladder to reach the upper echelons of participation (i.e., Metafy, ProGuides, Skill Capped). However, it is unclear what the objective difference is between experts and novices in their search for affordances. Research can use eye-tracking technology through the vision-in-action paradigm (Vickers, 1996), to allow an ecological investigation of attunement to visual information. Furthermore, reflective techniques such as the Think Aloud (Nicholls and Polman, 2008; Whitehead et al., 2016) approach can be used with the athletes to understand why they are using certain affordances to guide behavior and decision-making. Studies like this can better understand how esport athletes develop, and how that process can be streamlined.

### 5.2. Shared affordances and (re)organization in team esports

A large majority of esports performance is conducted in high-performing team environments. Successful teams rely on coordinative and synergistic actions to be successful (Dutt-Mazumder et al., 2011). Sports teams through an ecological lens have now been incorporated as complex adaptive systems (CAS; Arrow et al., 2000; Davids et al., 2005). Complex adaptive systems can adapt their behavior to one another as they interact with one another to achieve a common goal (Deneubourg and Goss, 1989). During performance as the game evolves, teams are required to problem solve and (re) organize themselves to maintain stability, and for synergies to perform two or more agents must perceive the same course of action from a shared affordance. Following a similar methodology as the study proposed in section “Perceptual attunement between novices and experts in esports”, eye tracking technology can be used to understand the visual search of the players, and in moments where multiple players are identifying similar information, that can be defined as shared affordances. Furthermore, qualitative techniques, such as the Applied Cognitive Task Analysis (ACTA), are used to understand how individuals/teams approach certain situations (Price et al., 2021). A study like this can provide invaluable information toward understanding how high-performing esports team coordinates their actions, how coordination be developed, and what makes team successful.

### 5.3. The application of the constraint-led approach to coaching in esports

Constraints are properties that can affect and shape behavior (Renshaw et al., 2019). CLA is a tool that coaches can use to manipulate certain affordances (esport dependant) to promote specific affordances in the environment. CLA is yet to be studied and applied in the novel context of esport performance and training. As previous literature (see section “Skill development and current esports research”) has already indicated the need for higher quality practice environments, CLA could provide suitable answers to more effective learning and development of esports athletes.

## 6. Conclusion

The purpose of this perspective paper was to conceptualize the application of an ecological and constraints-led approach within a novel context such as esports. By identifying the various principles of ecological dynamics (i.e., affordances and constraints) a better understanding can be developed of esports performance and can be used to inform training methods in professional or grassroots contexts. It is also plausible that such studies can uncover the knowledge that can be related back to traditional sporting contexts (e.g., football, basketball, rugby). Hopefully, this paper will encourage more skill acquisition researchers to look to understand the field of esports as it continues to grow in participation and competition.

## Data availability statement

The original contributions presented in the study are included in the article/Supplementary material, further inquiries can be directed to the corresponding author.

## Author contributions

KB: writing original draft preparation. KB, MT, MW, and RP: writing—review and editing and project administration. MT, MW, and RP: supervision. All authors contributed to the article and approved the submitted version.

## Conflict of interest

The authors declare that the research was conducted in the absence of any commercial or financial relationships that could be construed as a potential conflict of interest.

## Publisher’s note

All claims expressed in this article are solely those of the authors and do not necessarily represent those of their affiliated organizations, or those of the publisher, the editors and the reviewers. Any product that may be evaluated in this article, or claim that may be made by its manufacturer, is not guaranteed or endorsed by the publisher.

## Appendix 1

How CLA can be implemented in Esports. A comparison between CS:GO & League of Legends.

**Table 1:**

Training activity	EDP	Why?
Counter-strike: Global offensive		
Retake game mode	Repetition without repetition	High repetition of retake scenario for the counter-terrorist (CT) side Each round will be highly variable with the same objective Round starts with the bomb planted giving CT side 40 s to secure the site and defuse
Map boundary modification	Constrain to afford–environment constraint	Limiting the map area to one bombsite, therefore only certain points of entry & areas to control Developing creativity in lines of sight for the terrorist (T) side as the CT team has fewer rotations to make and can set up in corners More thorough search and slower advancements through areas of the map (i.e., chokeholds)
Headshot only command	Constrain to afford–task constraint	Kills only come from headshots Developing cross-hair placement and recoil control Better tracking of moving opponents and trigger discipline Finding advantageous lines of sights
League of Legends		
Training activity	EDP	Why?
No communication–scrim	Constrain to afford–environment constraint	Developing implicit coordination using the in-built ping system More effective use of the minimap to gather information (i.e., location of teammates and opposition)
No vision–Scrim	Constrain to afford–task constraint	Using deductive reasoning and explicit communication to predict the future actions of the opposition Ensuring proper support when entering opposition territory to control essential areas or support the designated warder (support) to place vision
Howling Abyss Map (ARAM Game Mode)	Repetition without repetition	Map consists of a singular lane, promoting constant action High repetition of team fight situations, developing team fight execution
